# Firing Behavior and Network Activity of Single Neurons in Human Epileptic Hypothalamic Hamartoma

**DOI:** 10.3389/fneur.2013.00210

**Published:** 2013-12-27

**Authors:** Peter N. Steinmetz, Scott D. Wait, Gregory P. Lekovic, Harold L. Rekate, John F. Kerrigan

**Affiliations:** ^1^Comprehensive Epilepsy Center, Barrow Neurological Institute, St. Joseph’s Hospital and Medical Center, Phoenix, AZ, USA; ^2^Carolina Neurosurgery and Spine Associates, Levine Children’s Hospital, Carolinas Medical Center, Charlotte, NC, USA; ^3^House Ear Institute, University of Southern California School of Medicine, Los Angeles, CA, USA; ^4^The Chiari Institute, Hofstra Northshore LIJ College of Medicine, Great Neck, NY, USA; ^5^Division of Pediatric Neurology, Barrow Neurological Institute at Phoenix Children’s Hospital, Phoenix, AZ, USA

**Keywords:** hypothalamic hamartoma, epilepsy, single unit, bursting, synchrony, microelectrode

## Abstract

**Objective:** Human hypothalamic hamartomas (HH) are intrinsically epileptogenic and are associated with treatment-resistant gelastic seizures. The basic cellular mechanisms responsible for seizure onset within HH are unknown. We used intra-operative microwire recordings of single neuron activity to measure the spontaneous firing rate of neurons and the degree of functional connection between neurons within the tumor.

**Technique:** Fourteen patients underwent transventricular endoscopic resection of HH for treatment-resistant epilepsy. Prior to surgical resection, single neuron recordings from bundled microwires (total of nine contacts) were obtained from HH tissue. Spontaneous activity was recorded for two or three 5-min epochs under steady-state general anesthesia. Off-line analysis included cluster analysis of single unit activity and probability analysis of firing relationships between pairs of neurons.

**Results:** Altogether, 222 neurons were identified (mean 6 neurons per recording epoch). Cluster analysis of single neuron firing utilizing a mixture of Gaussians model identified two distinct populations on the basis of firing rate (median firing frequency 0.6 versus 15.0 spikes per second; *p* < 10^−5^). Cluster analysis identified three populations determined by levels of burst firing (median burst indices of 0.015, 0.18, and 0.39; *p* < 10^−15^). Unbiased analysis of spontaneous single unit behavior showed that 51% of all possible neuron pairs within each recording epoch had a significant level of firing synchrony (*p* < 10^−15^). The subgroup of neurons with higher median firing frequencies was more likely to demonstrate synchronous firing (*p* < 10^−7^).

**Conclusion:** Hypothalamic hamartoma tissue *in vivo* contains neurons which fire spontaneously. The activity of single neurons is diverse but distributes into at least two electrophysiological phenoytpes. Functional linkage between single neurons suggests that HH neurons exist within local networks that may contribute to ictogenesis.

## Introduction

Hypothalamic hamartomas (HH) are congenital, non-progressive tumors of the ventral hypothalamus. HH that attach in the region of the mammillary bodies are associated with gelastic seizures, which usually begin during infancy and are treatment-resistant to conventional anti-epilepsy drugs (AEDs) (1, 2). A disabling clinical course, with the emergence of multiple seizures types, along with cognitive and behavioral decline, occurs in 50% of patients (3, 4). Surgical resection of the HH is a treatment option for patients who fail to respond to AEDs (5–10).

Recordings from surgically implanted intracranial electrodes have demonstrated that gelastic seizures originate in the HH (11–13). However, the cellular mechanisms responsible for intrinsic epileptogenesis of HH tissue are unknown. Study of surgically resected HH tissue has provided several observations relevant for a model of ictogenesis. HH tissue has at least two neuronal phenotypes: small (soma diameter usually <16 μm) and large (>16 μm) HH neurons (14–16). Microelectrode patch-clamp recordings have shown that small HH neurons have intrinsic pacemaker-like activity and fire spontaneously even in the absence of synaptic inputs (16, 17). Small HH neurons are abundant (approximately 90% of all HH neurons), express glutamic acid decarboxylase (GAD), and have an interneuron-like phenotype (15, 16). Large HH neurons (approximately 10% of all neurons) have the functionally immature property of depolarizing and firing in response to GABA, likely as a result of reversal of the transmembrane chloride gradient (17–19). High-density multielectrode field recordings of perfused HH tissue slices show network phenomenon, such as high-frequency oscillations (20). Collectively, these findings suggest a model for HH ictogenesis in which hypersynchrony of GABA-expressing interneurons paradoxically excite large HH projection-type neurons (21, 22).

All of these observations, however, are obtained from resected tissue slices, where the natural connections between cells have been disrupted (23). We wished to study the activity profile of HH neurons *in vivo*, prior to surgical resection, when all network connections are intact. By inserting bundled microwires into the HH through a surgical endoscope, we have been able to record single neuron activity. These recordings have allowed us to address three questions regarding the electrophysiology of these neurons *in vivo*: (1) is there evidence for spontaneous firing of HH single units? (2) Do HH neurons have burst-like firing patterns? (3) Is there evidence for functional connectivity of HH single unit firing activity, or are HH neurons electrically isolated from one another?

## Materials and Methods

All experiments were performed after informed consent was obtained under protocols approved by the Institutional Review Board for Human Research at Barrow Neurological Institute, St. Joseph’s Hospital and Medical Center, Phoenix, AZ, USA.

### Patient profile

Intra-operative *in situ* microwire single neuron recordings were obtained from 14 patients (9 females, 64%) undergoing surgical resection of HH at Barrow Neurological Institute between January 2008 and February 2010. Mean age at the time of surgery was 15.7 years (range 2.2–40.2 years). All patients had treatment-resistant seizures refractory to at least three AEDs.

Of the 14 patients, 10 (71%) experienced the onset of seizures before the age of 1 year, including 7 (50%) whose seizures began during the first month of life. At surgery, 13 (93%) patients had multiple daily seizures. The remaining patient had at least one seizure per week. At the time of resection, 3 patients (21%) had only gelastic seizures while 11 (79%) had multiple seizure types. At some time during their clinical course, all patients (100%) had gelastic seizures. Patients were taking the following AEDs at the time of surgery: carbamazepine (three patients), clobazam (1), clonazepam (1), lamotrigine (2), levetiracetam (7), oxcarbazepine (3), phenobarbital (1), phenytoin (1), tiagabine (1), topiramate (2), valproic acid (1), zonisamide (2), no AEDs (1). At surgery 11 patients (79%) were taking more than one AED.

Four patients (29%) had mental or developmental retardation (full-scale intelligence quotient or estimated developmental quotient <70). Five patients (36%) had a history of central precocious puberty. No patients had Pallister–Hall syndrome or any other syndromes. Two cases (14%) had previously undergone Gamma Knife radiosurgery, and one case (7%) had previously undergone subtotal resection of the HH lesion. Based on Delalande’s classification of HH anatomic subtypes (24), eight patients had Type II (57%), four had Type III (29%), and one had Type IV (7%) HH.

Hypothalamic hamartomas were resected via the transventricular endoscopic approach under anesthesia using previously described methods (25). The mean HH lesion volume was 0.8 cm^3^ (range 0.1–4.8 cm^3^). In all cases, pathological analysis confirmed the diagnosis of HH.

### Recording single neuron activity

Before resecting the HH, we recorded the activity of single neurons within the hamartoma, using techniques previously described in Ref. (25). Briefly, nine 38-μm diameter platinum-iridium microwires were inserted through the working channel of the endoscope. The microwires were connected to a custom headstage amplifier providing 400× gain of the difference in extracellular voltage between eight of the microwires and a ninth microwire used as the reference. The output of this amplifier was further amplified by 16× and bandpass filtered between 0.5 and 10000 Hz. The signal was then connected to a digital system that recorded the voltage differences, sampling at a rate of 29412 Hz. Two or three 5-min recording epochs were obtained from each patient. Between each recording epoch, the microwire bundle was advanced 0.5–1 mm.

Waveform events containing putative action potentials (1.1 ms) were captured around the time of large voltage deviations in the recorded extracellular potential (outside ±2.8 SDs for the entire recording epoch). The events for each channel were sorted into clusters of similar waveform shape using the program KlustaKwik (http://klustakwik.sourceforge.net) and methods previously described in Ref. (26) to isolate the activity of single neurons. Based on the shape of the waveform, interspike interval histogram, and power spectrum of the spike occurrence times (25), clusters were graded as single unit (neuron) activity or as noise.

### Data and statistical analysis

To measure the mean firing rate of recorded neurons, we computed the average number of spikes per second (sp/s) during the entire recording session. To measure the tendency of neurons to fire in bursts, we computed the burst index (*B*_isi_) according to the following formula:

$$B_{\text{isi}}=\frac{\text{number of interspike intervals}<10\text{ ms}}{\text{number of interspike intervals}\geq 10\text{ ms}},$$

where isi = interspike interval. *B*_isi_ has been validated for studying *in situ* firing behavior of single neurons in human epileptic temporal lobe (27). All statistical calculations were performed using the R statistics program (28). To test the hypothesis that HH neurons fire within functional networks, we examined the degree to which every possible pair of identified neurons within a single recording session demonstrated functional linkage (synchronous or near-synchronous firing) using a method adapted from Roy et al. (29). To avoid recording the same neuron firing event from two different recording wires, we first deleted the second spike of any pair in which the interspike interval was <100 μs. This boundary time was chosen to eliminate spikes which were being recorded in two different channels within three sample intervals (1/29412 of a second) of each other. Such coincidence is likely due to recording the same unit in multiple channels, rather than two units linked by a biological process such as synaptic transmission.

Each 5-min recording epoch was divided into 2-s segments. For each 2-s segment, a cross-correlogram between the firing times of each neuron in the pair was computed by recording the number of action potentials within 1/1024 s bins equally spaced in the epoch for each neuron; spectral methods were then used to calculate the cross-correlation between these two discrete sequences (30). The resulting cross-correlograms for each 2-s segment were averaged.

The significance of the peak in the averaged cross-correlogram near 0 delay was assessed using a permutation test. In this technique, each of 200 synthetic samples was chosen by randomly shuffling the order of the epochs of the second neuron of a pair. The averaged cross-correlogram was then computed as above. The empirical distribution of the height of the peak in the averaged cross-correlogram near 0 delay was used to determine the significance of the height of the peak obtained when the cross-correlogram was computed without shuffling (31). Because the average firing rates of each neuron in the pair were unaffected by the shuffling, the significance level determined in this manner was independent of firing rate.

The statistical significance of separation between two modes in the distribution of the firing rates or burst index was assessed with Hartigan’s dip-test (32), which assesses how large the differences are between the peaks in the distribution at the modes and the best fitting unimodal distribution. To characterize the multi-modal distributions of firing rate and burst index, we fit the distributions with a mixture of Gaussians model (33, 34) and report the mean and median of the populations determined by assigning each cluster to the model Gaussian distribution with the highest likelihood. The number of Gaussians used was chosen by successively adding a Gaussian to the model until there was no further significance improvement in model fit, based on the Akaike information criterion (35).

To determine whether the fraction of neurons with high versus low firing rates is related to the fraction of neurons which are in pair with a significant level of synchronous firing, we used Fisher’s exact test (36), which computes the likelihood of obtaining the observed fractions of these properties assuming the two fractions are independent of one another. To determine whether the fraction of neurons with significant levels of synchronous firing differed between patients, we used Pearson’s chi-square test of independence (36), which determines the likelihood of obtaining the observed fractions assuming they are equal for all patients.

To account for the effects of varying anesthetic conditions, we constructed a set of generalized linear models for each neuron where the dependent variable, firing rate, was expressed as a linear combination of these independent variables. For continuous independent variables, such as temperature, the variable could have a linear multiplicative effect on firing rate. For categorical variables, such as whether midazolam was used during induction, the presence of the category provided an additive effect on firing rate. The significance of adding each independent variable to the model, without interaction, was assessed using an *F*-ratio test. The *p*-values obtained from these multiple tests were corrected by controlling the false discovery rate to 0.2 (37).

## Results

### Neuronal firing rates

**Figure 1:**
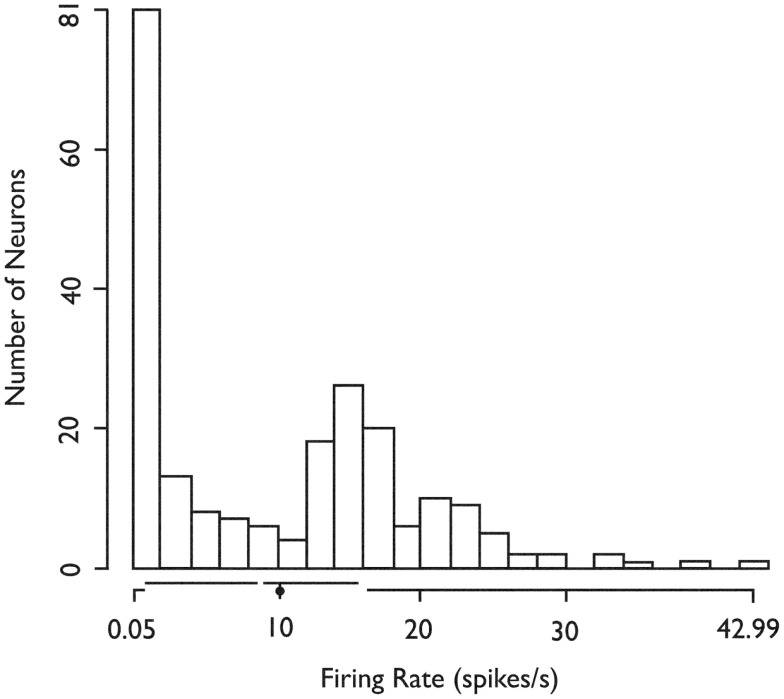
**Histogram of the *in situ* firing rates of all 222 (HH) neurons**. *X*-axis. Limits of axis show minimum and maximum firing rates. Offsets in axis line indicate first and third quartiles. Break in axis line indicates median. Gray dot indicates mean. *In situ* firing rates for HH neurons distribute into at least two classes (fast-firing and slow-firing units). *Used with permission from Barrow Neurological Institute*.

We recorded the firing activity of 222 neurons in 37 5-min recording epochs from 14 patients (Table 1). For all included neurons, the mean firing rate was 9.9 sp/s (SD = 9.3 sp/s, median value 8.8 sp/s). The mean firing rate over the entire recording epoch for each neuron is shown in Figure 1. Based on Hartigan’s dip-test (32), the null hypothesis that there was only one mode in this distribution was rejected (*p* < 10^−5^). Fitting this distribution with a mixture of Gaussians model produced two classes: those with a mean firing rate <2 sp/s (slow-firing; median 0.6 sp/s) and those with a mean firing rate ≥2 sp/s (fast-firing; median 15.0 sp/s).

**Table 1:** **Number of neurons recorded for each subject**.

Subject	Recording epoch	Neurons per epoch
HH2114	2	1
	3	5
HH2115	2	4
	3	1
HH2116	2	3
	3	13
HH2128	1	7
	2	14
	3	12
HH2132	1	5
	2	9
	3	8
HH2133	1	3
	2	3
HH2135	1	3
	2	1
HH2138	1	7
	2	4
	3	10
HH2139	1	3
	2	6
	3	2
HH2140	1	12
	2	12
	3	8
HH2144	1	1
	2	3
	3	1
HH2145	1	1
	2	7
	3	8
HH2147	1	9
	2	8
	3	7
HH2149	1	6
	2	13
	3	2

### Firing in bursts

**Figure 2:**
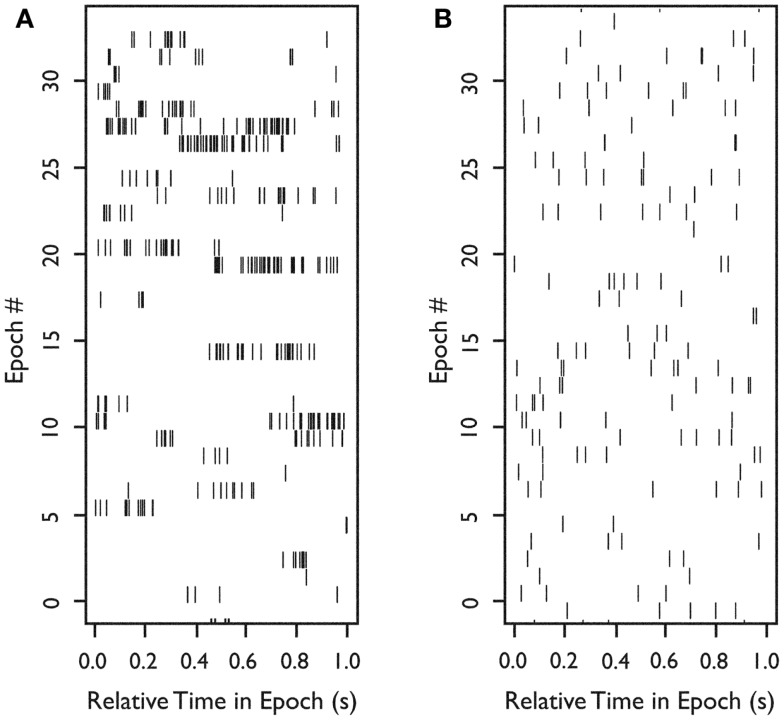
**Firing of two neurons during 33 s of continuous *in situ* recording**. Raster plots of spike occurrence times. *X*-axis: relative time of spike occurrence in each 1-s interval; *y*-axis: number of 1-s sequential intervals within the recording epoch. **(A)** Raster for a neuron with a tendency to burst (*B*_isi_ = 0.9). **(B)** Raster for a neuron with more uniform firing (*B*_isi_ = 0.039). *Used with permission from Barrow Neurological Institute*.

To examine burst-firing behavior, we computed *B*_isi_ for all 222 neurons. For neurons with a high *B*_isi_, firing was analyzed in 1-s intervals throughout each 5-min recording epoch. Figure 2A shows an example of the firing of a neuron with a tendency toward burst firing, whereas Figure 2B shows an equivalent time period for a neuron with more uniform firing throughout the recording session.

**Figure 3:**
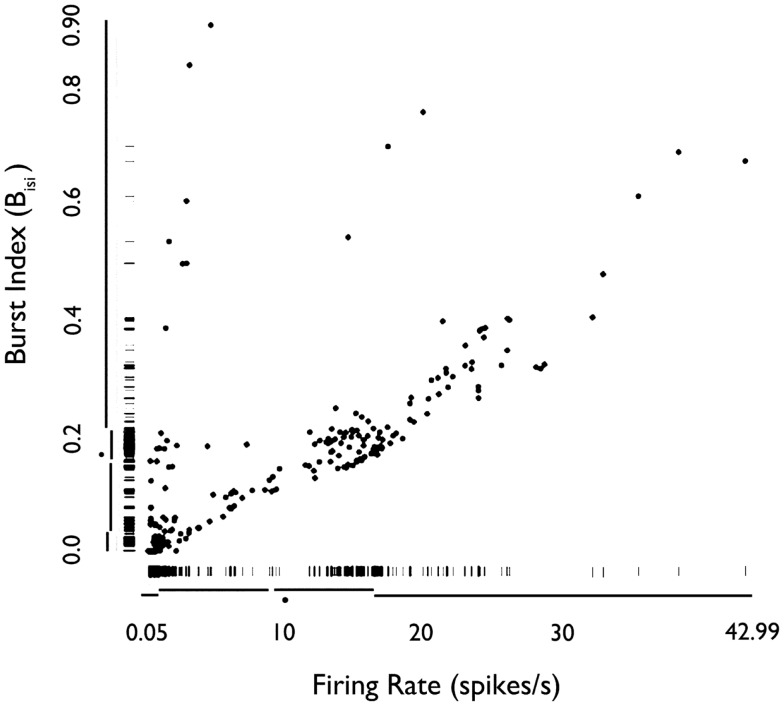
**Rug plot of mean firing rate of HH neurons versus burst index (*B*_isi_)**. Fast-firing units (mean firing rate >2 sp/s) scored higher on the burst index. Conversely, slow-firing units (mean firing rate <2 sp/s) were associated with lower scores on the burst index. However, units with relatively slow-firing features and bursting tendencies were also observed. *Used with permission from Barrow Neurological Institute*.

We examined how the mean firing rate and *B*_isi_ were jointly distributed for all 222 neurons (Figure 3). Based on Hartigan’s dip-test (32), the null hypothesis that there was only one mode in the distribution of *B*_isi_ was rejected (*p* < 10^−15^). Fitting this distribution with a mixture of Gaussians model provided a best fit with three classes, with median burst indices of 0.015, 0.18, and 0.39. These three groups are visible in the marginal distribution along the *y*-axis of Figure 3. The joint distribution shows that neurons with higher firing rates tended to have higher bursting, as measured by *B*_isi_ (Pearson’s ρ = 0.66). Consequently, these two firing phenotypes may be interdependent. However, slow-firing neurons with bursting behavior were observed (Figure 3).

### Synchronous firing of pairs of neurons

**Figure 4:**
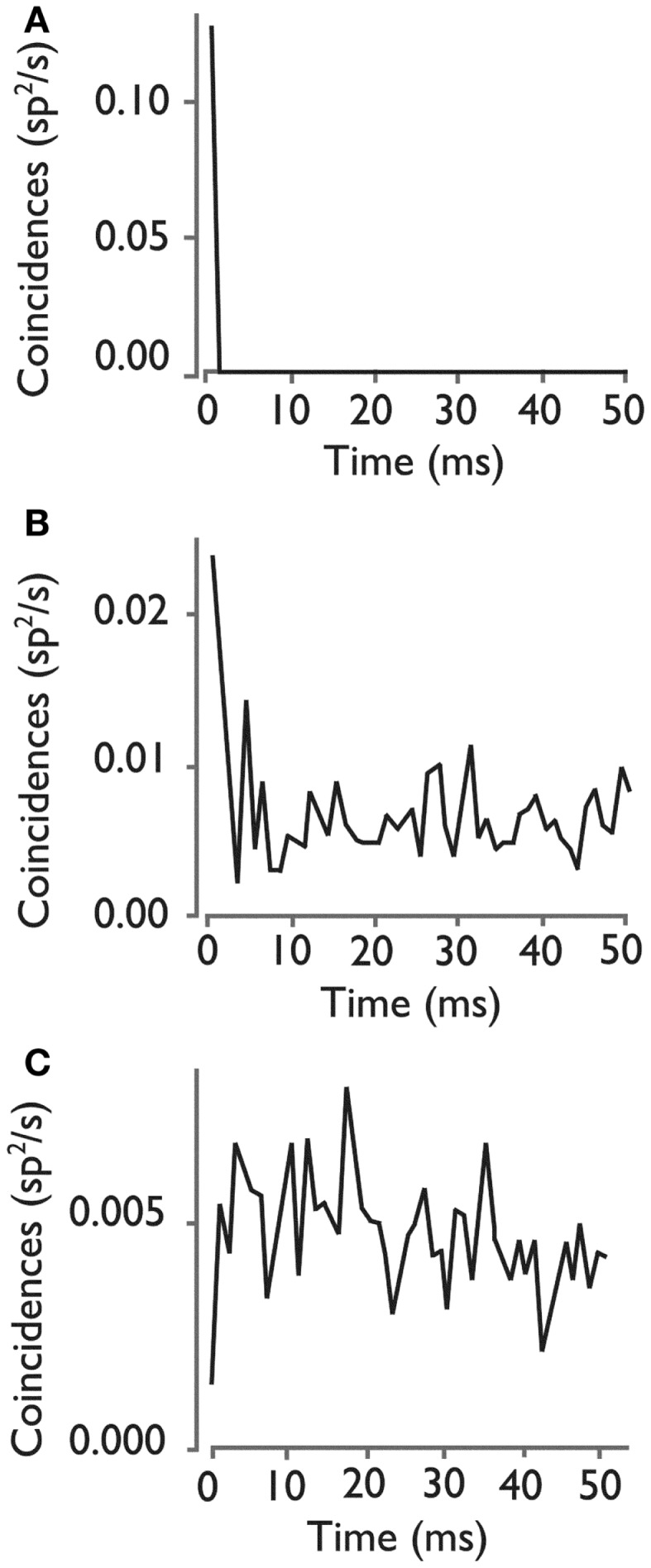
**Cross-correlograms for three pairs of HH neurons**. **(A)** Rate of coincident firing as a function of the delay between firing for a neuron pair with a significant high level of synchronous firing. **(B)** Cross- correlogram for neuron pair with an insignificant level of synchronous firing, showing the types of peaks that can randomly occur in a cross-correlogram. **(C)** Cross-correlogram for a pair of neurons with a significant inhibition of synchronous firing at 0 delay. *Used with permission from Barrow Neurological Institute*.

To study the extent of synchronous firing between pair of neurons, we examined the cross-correlograms of all possible neuron pairs during each recording epoch. Figure 4A illustrates a neuron pair that was more likely to fire together relative to random expectations (*p* < 0.05, bootstrap test), as shown by a peak near 0 delay in the cross-correlogram. Figure 4B illustrates a representative neuron pair with a random firing relationship. In some cases, neurons in a pair were less likely to fire together, as shown by a negative peak (Figure 4C), suggested functional inhibition within the network.

**Figure 5:**
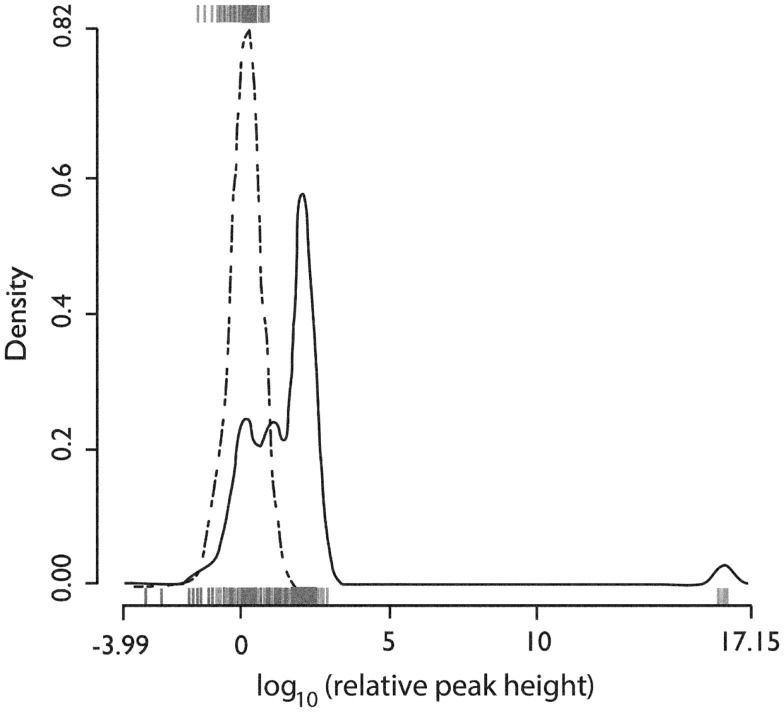
**Distributions of the relative height of the cross-correlogram peak at 0 delay for all recorded pairs of neurons**. The distribution for positive peaks is shown by the rug plot on the lower *x*-axis with an estimated density shown by the solid line (Gaussian kernel with SD = 0.25). The distribution for negative peaks is shown by the rug plot on the upper *x*-axis with estimated density shown by the dotted line (Gaussian kernel with SD = 0.25). *X*-axis is the base 10 logarithm of the absolute value of the peak height relative to the standard deviation of cross-correlogram values in a window from 1 to 25 ms delay. *Used with permission from Barrow Neurological Institute*.

Figure 5 shows the distribution of the heights of the cross-correlogram peaks at 0 delay for all possible 813 pairs of neurons derived from each of the 37 recording epochs. Of these, 417 (51%) had a peak in the cross-correlogram at 0 delay. This finding was significantly different from that expected by chance (*p* < 10^−15^, binomial test). Within this subgroup of 417 neuron pairs, 281 (67%) were positive peaks, indicating an increased level of firing together in time.

**Figure 6:**
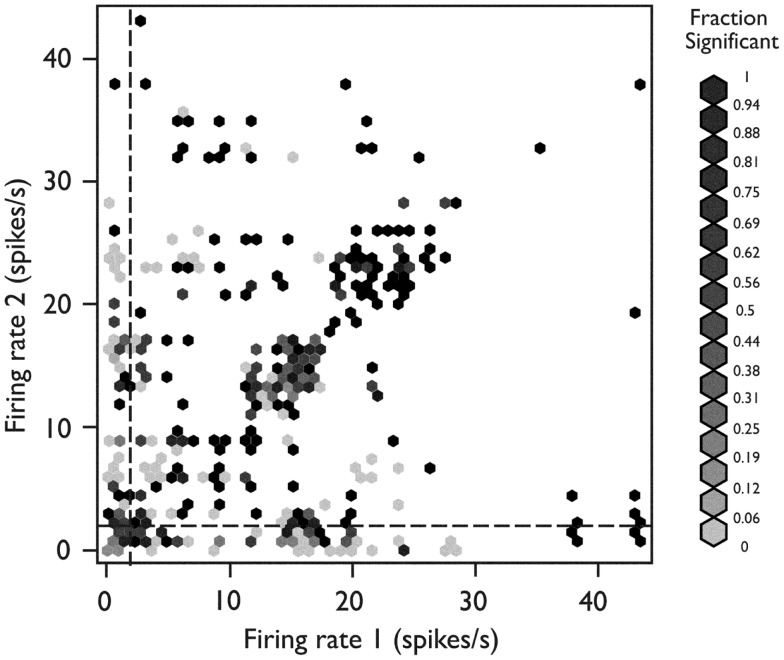
**Relationship between network behavior and mean firing rate**. Each neuron pair is plotted according to the mean firing rate of neuron 1 (*x*-axis) and neuron 2 (*y*-axis). The relative strength for synchronous firing behavior for each pair is grayscale coded within the hexagon representing that pair (darker hexagon indicates a pair with more synchronous firing behavior within the recording epoch). The neuron pairs in which both neurons were members of the fast-firing class (mean firing rate >2 sp/s) were more likely to exhibit synchronous firing behavior (*p* < 10^−15^, Fisher’s exact test). *Used with permission from Barrow Neurological Institute*.

To identify any relationship between the firing behavior of a single neuron and the likelihood that it would be engaged in synchronous firing with other neurons (Figure 6), we plotted each neuron pair at the *x*-*y* coordinate corresponding to the mean firing rate of neuron 1 (*x*-axis) and neuron 2 (*y*-axis). Each pair was color coded for its tendency to fire in a synchronous manner. When both neurons were fast-firing (mean firing rate >2 sp/s), they were more likely to exhibit synchronous firing behavior (*p* < 10^−7^, Fisher exact test). The peaks in cross-correlograms tended to be higher for faster mean firing rates, but the bootstrapped significance test used in these analyses compensated for this relationship. Thus, these results indicate that neurons in the fast-firing class are also more likely to be synchronized within the functional network.

To determine whether different HH lesions (different patients) were likely to show the same levels of single unit firing synchrony, we analyzed the number of neuron pairs with and without synchronous firing for each patient. Based on Pearson’s chi-square test, the null hypothesis that there was no difference in the fraction of pairs with synchronous firing between patients was rejected (*p* < 2 × 10^−16^). Likewise, the results of *in situ* recordings from different locations within the same HH lesion were non-uniform (*p* = 0.025). This finding indicates regional heterogeneity of network activity within the HH, consistent with neuronal clustering.

### Single unit activity by patient and anesthesia

**Figure 7:**
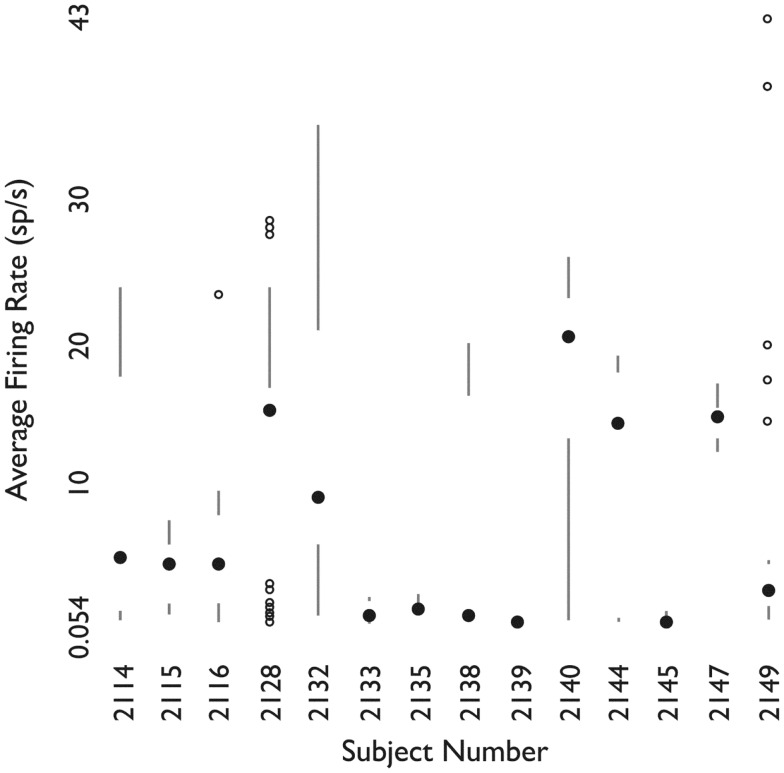
**Firing rates of neurons grouped by patient**. For each patient, shown as one column, the filled circle shows the median firing rate. The bars above (below) extend from the third (first) quartile to the data point furthest from the median not more than 1.5 times the inter-quartile range from the median. Points further from the median are shown as small open circles. *Used with permission from Barrow Neurological Institute*.

Figure 7 shows the distribution of firing rates for HH neurons grouped by individual patient. Given our finding of two classes of neurons based upon mean firing rate, we wanted to determine whether these classes might reflect independent variables, such as differences in anesthesia, which was not under experimental control. Table 2 shows the variables which were included in a generalized linear model of anesthesia-related effects, whether they were significant after correction for false discoveries, and if significant, the fraction of variance of the firing rates which was accounted for by that variable. While a number of these variables have a significant effect on firing rates, all of the significant variables combined accounted for only 15% of the total variation in firing rates. The residual variations in firing rates, after accounting for the anesthesia-related factors in this linear model, still showed evidence of two or more classes of neurons (Hartigan’s dip-test, *p* < 0.05), reinforcing the conclusion that two classes of neurons with different firing rates are present.

**Table 2:** **Effects of anesthesia on neuronal firing rates**.

Variable	*p*-Value (*F*-ratio test)	Significant after correction	Fraction of variance	Effect
Use of fentanyl at induction, at intervals, or not at all	0.70	No		
Patient temperature	0.11	No		
Gas anesthetic containing air, NO, or neither	0.02	No		
Use of decadron	0.33	No		
Sevoflurane dose	0.017	Yes	0.022	Mean increase of firing rate by 31 sp/s per percent of sevoflurane in inhaled gas
Use of midazolam	0.013	Yes	0.024	Mean decrease of firing rate by 11 sp/s with use of midazolam at induction
Use of propofol at induction	0.0005	Yes	0.047	Mean increase of firing rate by 2.7 sp/s with use of propofol at induction
Time since last change in anesthetic	0.00009	Yes	0.06	Mean increase of firing rate by 0.16 sp/s per minute of time since last change

### Evolving single unit discharges

**Figure 8:**
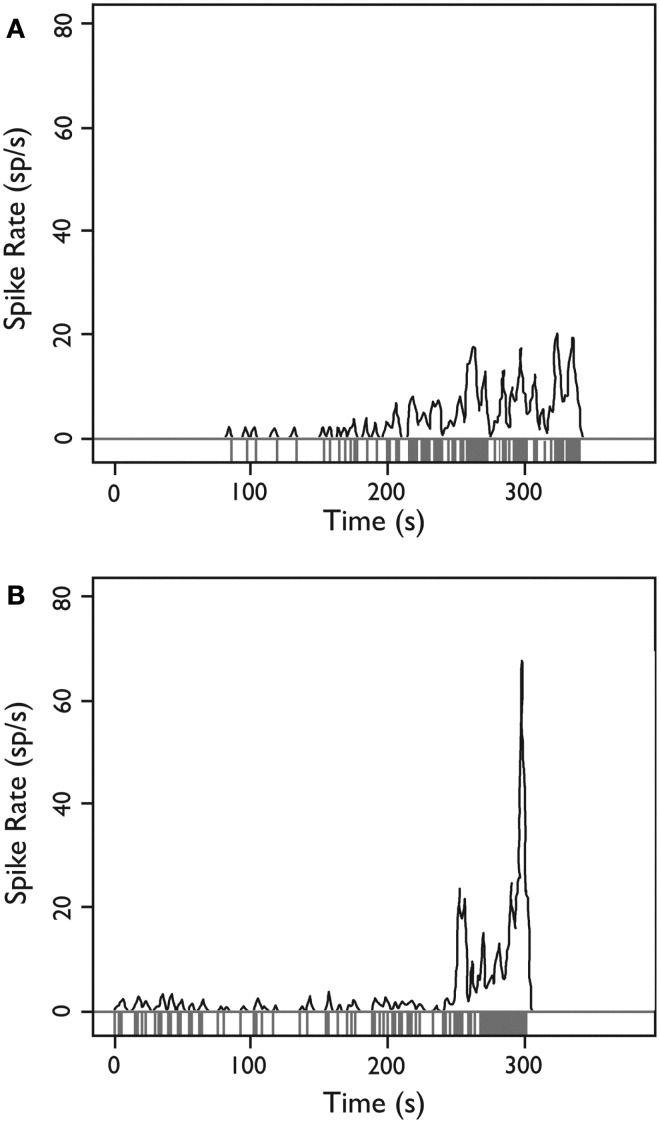
**Firing behavior over time for two HH neurons**. **(A,B)** Each neuron demonstrates a sudden departure from baseline behavior with an evolving discharge of firing activity, potentially consistent with a local ictal discharge. *Y*-axis: firing rate in spikes per second estimated with a Gaussian kernel with SD = 1 s; *x*-axis: time during recording epoch. *Used with permission from Barrow Neurological Institute*.

When the firing rate averaged over 1-s intervals throughout the experiment was examined, we occasionally observed neurons (8 of 222; 3.6% of all observed single units) whose firing rate departed from baseline with a brief evolving discharge. Two neurons illustrating this behavior are shown in Figure 8.

## Discussion

### Research objective

Our goal was to measure firing properties of HH neurons in an intact network in order to constrain possible models of ictogenesis. Our findings are: (1) there are two classes of neurons in the HH tumor, separated by having spontaneous firing rates above or below 2 sp/s, and (2) that faster firing neurons are more likely to be involved in pairs of neurons which fire synchronously.

### Single unit (neuron) phenotypes

Prior studies of single unit firing in HH tissue *in vitro* have identified two populations of neurons in human epileptic HH, recorded under several different conditions (Table 3). Utilizing patch-clamp techniques on acutely dissociated single neurons (that is, neurons stripped of all synaptic contacts), Wu and colleagues described highly regular, pacemaker-like firing activity (mean firing rate 10.5 sp/s) from small HH neurons (16, 38). These findings suggest that this activity is an intrinsic membrane property of the small HH neuron (19). Conversely, in surgically resected, perfused HH tissue slices, the mean firing rate was 6.6 sp/s for small HH neurons (17). Furthermore, Kim and colleagues observed variability in the intrinsic pacemaker-like firing behavior (*n* = 64 small neurons, regular firing 63%, irregular firing 28%, burst firing 9%), suggesting that firing behavior related to intrinsic membrane features is influenced by partial preservation of the network present in the tissue slice (17).

**Table 3:** **Firing behavior. of human HH neurons under different recording conditions**.

Reference	HH tissue condition	Recording platform	Small HH neurons	Large HH neurons	Unknown HH neuron type
Wu et al. (16)	Acutely dissociated single cells	Patch clamp	10.5 ± 0.8 Hz	Not reported	
Wu et al. (38)	Acutely dissociated single cells	Patch clamp	Range 7–14 Hz	Not reported	
Wu et al. (19)	Acutely dissociated single cells	Patch clamp	9.9 ± 0.8 Hz	3.7 ± 0.4 Hz (“when firing”)	
Kim et al. (17)	Perfused tissue slice	Patch clamp	6.6 ± 1.0 Hz	“Quiescent”	
Steinmetz et al. (this report)	*In situ* intra-operative (before surgical resection)	Bundled microwire field recording			Mean 9.9 Hz
					First population peak 0.6 Hz
					Second population peak 15.0 Hz

These findings raise the question of whether the two classes of neurons identified *in vitro* correspond to neural firing in the intact tumor as described here. A comparison of these *in vitro* results with the present recordings suggests that disrupting the network during resection may change the properties of neural firing. The two classes of neurons identified in the present study have median firing rates of 15.0 and 0.6 sp/s, both of which could correspond to the firing of small neurons reported in *in vitro* recordings (Table 3) [mean firing rate of 6.6 sp/s (17)], rather than the nearly quiescent firing of the large neurons observed *in vitro*.

Based on the relative abundance of small HH neurons and the tendency of large HH neurons to be quiet in slice recordings, we posit that the fast-firing and slow-firing units identified here are both expressions of small HH neuron activity, reflecting different activity phenotypes within the native (*in situ*) network.

As our analysis showed, while it may not be possible to experimentally control administration of anesthesia in human studies of this sort, variations in anesthetic conditions accounted for only 15% of the variation of firing rates amongst neurons, and two classes of firing were present even when these conditions were controlled for.

### Synchrony of single unit (network) activity

When we examined the firing between pairs of neurons, we found that 51% of all possible pairs (identifiable single units captured during the same recording epoch) showed a significant level of synchronous firing, and that fast-firing neurons were more likely to be involved in synchronous pairs than were slow-firing neurons. This result suggests that functional connectivity between neurons is an important determinant of neural firing behavior within the tumor.

Based on these and prior observations we posit a model wherein the large projection-type neurons identified *in vitro* are relatively quiescent. The small neurons, by contrast, are frequently coupled to one another within the tumor in local networks. These connected small neurons fire at a faster rate, and synchronously. There is also a sub-population of small neurons which are functionally disconnected from these networks and which normally fire more slowly. We speculate that this functional network corresponds anatomically to the small neuron clusters that are present in HH tissue (15). Feedback loops within the local small neuron network may lead to increased synchrony of firing behavior, while projecting to the large excitatory neurons that are known to depolarize and fire in response to GABA (17–19). These projection-type neurons may, in turn, connect with normal brain circuits and lead to clinical seizures.

Given the lack of an animal model of HH, how can this model be tested within the constraints of clinical treatment and recording? One technique which could be very useful would be to label the location of the tips of the microwires within the tumor, either with electrolytic lesions or a fluorescent dye, and then resect the tissue around this point in as large a piece as possible. This could generate tissue where cell types for recordings could be identified histologically. Another potentially useful technique could be to use a fixed array of a larger number of microwires for recording neural activity to better characterize network activity as a whole.

### Evolving single unit discharges

In support of this model, we observed episodes of several seconds where neurons appeared to undergo a transformation to much higher firing rates (*cf*. Figure 8). Since the patient was under anesthesia, it is not possible to know whether this firing definitely corresponded to a clinical seizure, however, we note that macroelectrode depth wire recordings immediately before surgical resection have also shown seizure activity within HH tissue in patients under general anesthesia (39) and that seizure-like discharges have been reported in surgically resected HH tissue slices after provocation with 4-aminopyridine (4-AP) (20).

## Conclusion

Overall, the results of this study suggest there are three functional classes of neurons within human HH, consisting of large, relatively quiescent neurons and two sub-populations of smaller neurons. One sub-population is functionally connected and neurons within this sub-population tend to fire synchronously together, whereas the other sub-population fires more slowly and independently. These observations suggest a model of ictogenesis which involves increasing functional connectivity between small neurons generating episodes of high-frequency firing leading to seizures. This model may be tested by further experimental characterization of the network firing behavior within these tumors.

## Conflict of Interest Statement

The authors declare that the research was conducted in the absence of any commercial or financial relationships that could be construed as a potential conflict of interest.
